# Editorial Movements and Changes

**Published:** 1854-03

**Authors:** 


From the New York Medical Times.

*Editorial Movements and Changes. New York Journal of Pharmacy.*

This journal, which has just closed the second year of its existence, has passed from the editorship of Dr. McCready, and from under the patronage of the College of Pharmacy of this city, into the hands of Dr. Thomas Antisell, who is to be aided by Prof. Torrey, Dr. C. Enderlin, and Messrs. Edward N. Kent and Benjamin Canavan. The work is enlarged, and much improved in its typographical appearance, and is to give its readers 48 pages monthly, and of an increased size of page of type. With these advantages, and with the prospect of great improvement in the contents of the pages, we trust that it will prove worthy of our great city, and do much towards the promotion of science in its own field.

*Western Journal of Medicine and Surgery.*

A new series of this journal published at Louisville, Ky., and edited by Prof. L. P. Yandell, was commenced on the 1st of Jan. The old series, which numbered twenty-eight volumes, having been brought to an abrupt close by a fire in the printing office, in November last. The price has been reduced from five dollars to three dollars a year, for which amount eighty pages will be issued monthly. The character of the editor and the aid which he has at hand from his colleagues, give promise of a periodical of interest and of value.

*Journal of the Virginia State Medical Society.*

Dr. J. P. Atkinson has been appointed the financial editor of this Journal, to be issued under the auspices of the State Medical Society of Virginia. The assistant editors are Drs. Bolton, Lewis, Cabell, Thweat, Marx and C. Johnson.

The "Journal Committee" of the Society have purchased the *Stethoscope and Virginia Medical Gazette*, published at Richmond, and edited by Dr. Gooch, which is hereafter to be the organ of the Society. His confreres will regret to learn of the withdrawal of Dr. G. from a post which he filled with so much independence and industry.

From the Buffalo Medical Journal.

Dr. Samuel R. Hollingsworth has succeeded Messrs. Francis Gurney Smith, and John B. Biddle, M. D., in the editorial management of the Philadelphia Medical Examiner.

The Examiner has long sustained an excellent character among our periodicals, and we trust that Dr. Hollingsworth will well maintain the arduous *role* he has taken upon himself.

Dr. John Dawson has undertaken the editorship of the Ohio Medical and Surgical Journal, made vacant by the death of Prof. Howard. We wish him every success in his new field of labor.

The New Jersey Medical Reporter, one of the best of our exchanges, and which has increased in size and improved otherwise very rapidly under the editorial care of Dr. Joseph Parrish, has now been transferred to Dr. G. M. Butler. The cause of this change is Dr. Parrish's removal to Philadelphia. Dr. Parrish does not, however, entirely disconnect himself from the Reporter, and his monographs will occasionally appear in it. Just now he is writing an excellent series of papers on the "Change of Life" in women.

Dr. Dowler has assumed the editorial chair of the *New Orleans Medical and Surgical Journal.*

